# How do we estimate magnitudes? Validating the magnitude stroop task

**DOI:** 10.1371/journal.pone.0357108

**Published:** 2026-08-31

**Authors:** Michele Garagnani, Nitin Yadav

**Affiliations:** Center for Brain, Mind, and Markets, University of Melbourne, Melbourne, Victoria, Australia; University of Porto Faculty of Psychology and Educational Sciences: Universidade do Porto Faculdade de Psicologia e de Ciencias da Educacao, PORTUGAL

## Abstract

Humans have the ability to make spontaneous and rapid estimates of the approximate number of items they see, without explicitly counting them (e.g., estimating the number of fruits on a tree). However, most of our decisions are based on comparing symbolically represented numbers, such as Arabic numerals. Here, we validate a variant of the Stroop task, the “Magnitude Stroop,” which investigates differences in behaviour when numbers are represented as magnitudes versus symbols. In this task, stimuli are presented simultaneously as numerical symbols and magnitudes (e.g., the number 1 is represented by vertically aligned dots). In two studies (online *N* = 204 and offline *N* = 60), participants were asked either to select the stimulus with more dots or with the largest number. These two rules are occasionally equivalent but at times not, hence creating a possible Stroop interference effect. We find that the Stroop effect is stronger when participants are asked to select the stimulus with more dots and ignore the largest number, compared to when they should ignore the number of dots and select the largest number. These findings suggest that more research on how people estimate magnitudes across modalities is needed and we validate a method which could help investigations in this topic.

## Introduction

Humans, and many other animals, have the ability to make spontaneous and rapid estimates of the approximate number of items that they can see. This is often called the “numbersense” and it is particularly important because it is the basis of most mathematical skills. However, for most of our modern-life, economically-relevant decisions do not involve the estimation of how many items we see, but rely on comparisons between magnitudes which are symbolically represented, e.g., money using Arabic symbols. It is then natural to question whether our numbersense depends on the modality with which the numbers are represented (i.e., symbolic vs. non-symbolic). There is an extensive literature on this topic (e.g., see reviews by [1,2], which in this work we aim to contribute to by proposing a new method to study the potential interference different ways of representing magnitudes have on each others.

A large body of research has investigated differences in processing of stimuli across modalities (e.g., verbally vs. numerically [3] and in particular whether symbolic numbers (e.g., Arabic digits) and non-symbolic magnitudes (e.g., dot arrays) rely on a common, abstract representational system or on partly distinct codes that become linked through learning [4–9]. The received results are nuanced. Data is often consistent with the view that numerical symbols operate primarily as an associative system which often overshadows the relation between symbols and their quantity referents [8]. However, studies have also shown that a lot of nonnumerical cognitive processes might play a role in the tasks typically used to assess the interrelation between symbolic numbers and non-symbolic magnitudes (for a review [9], e.g., spatial–numerical associations [2], semantic priming [10], and attention [11]. Moreover, it is possible to dissociate numerosity and numerical values like when they are spatially separated, such that only task-relevant magnitudes determine behaviour [7], which speaks against fully integrated processing of symbolic and non-symbolic magnitudes. In this sense, studying the relation between numerical symbols and non-symbolic magnitudes is a surprisingly difficult and nontrivial process [8], which has lead to several investigations and the continuous development of new tasks aimed at exploring this topic.

**Fig 1:**
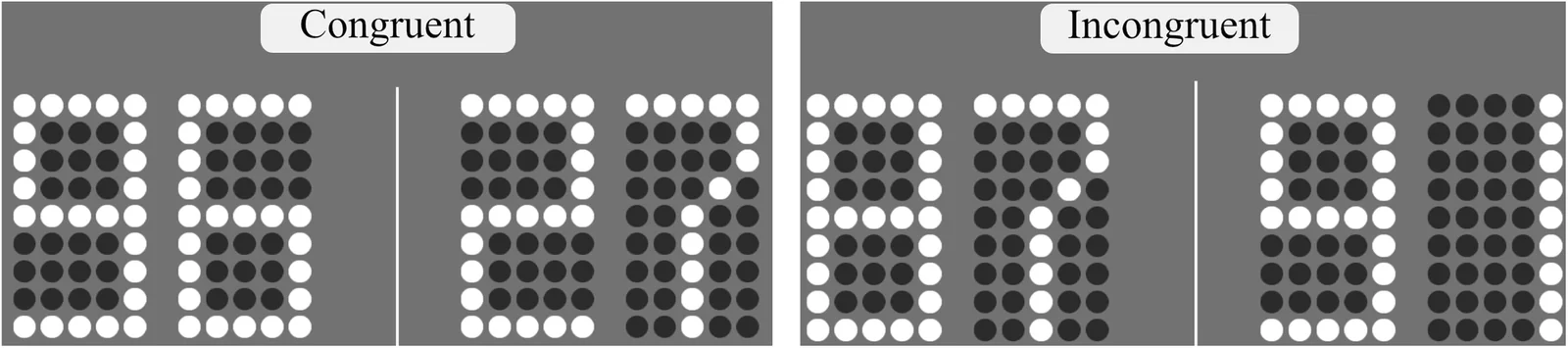
Example of the stimuli in the new Magnitude Stroop task for congruent stimuli (left-hand panel) and incongruent stimuli (right-hand panel).

With this idea in mind, we propose and empirically validate a variation of the Stroop task [12–15], which we call *Magnitude Stroop*. This new task relies on both dimensions of the stimuli being about magnitudes, symbolic and non-symbolic. That is, we present a series of dots of varying numbers arranged to form symbolic numbers in an Arabic format, see Fig 1 for examples of the stimuli. The intuition behind this task is that sometimes the number of dots composing the symbolic numbers correspond to largest number (congruent trials), but other times not (incongruent trials). Representing stimuli in this way allows us to independently vary two different dimensions of magnitudes at the same time. We then ask people to ignore one of the two dimensions and only consider the other. In practice, this corresponds to asking people sometimes to ignore which is the largest number and indicate which of the two stimuli contains more dots (DOT condition), while other times we ask people to ignore the number of dots and indicate which is the largest number (NUMBER condition). Crucially, both dimensions pertain to numbers and might interfere with each other. Comparing the performance and Stroop effect, i.e., the difference in the distribution of response times of correct choices between incongruent and congruent trials, between the two conditions tells us whether there is evidence for a common representation of numbers across modalities.

We hypothesise the presence of a Stroop-like effect in both conditions (DOT and NUMBER). In particular, observing a general Stroop effect in the MAGNITUDE Stroop task would suggest that there is at least some interference in how we process quantities compared to symbolic numbers, and hence that the two processes of estimating and comparing quantities share at least some common characteristics. Moreover, under which condition (DOT vs. NUMBER) the Stroop effect is more pronounced will provide evidence regarding which modality of representing magnitudes is more automatised.

The Magnitude Stroop is a modification of the original one, where both characteristics of the stimuli represented are numerical magnitudes. We are clearly not the first to use Stroop-like tasks to assess the processing of numbers [5,16,17]. Numerical Stroop [16] is the most widely used and involves numbers presented in different font sizes in order to create a congruent/incongruent effect compared to their magnitudes. The Numerical Stroop varies two physical dimensions, the classical numerical dimension and the font size. That is, the two dimensions create the potential incongruity by changing how salient one stimulus is compared to the other based on its font size independently of the magnitude of the displayed numbers [11]. Hence, this incongruity does not directly pertain to the numerical magnitudes of the displayed stimuli, but to the difference in saliency induced by the font size. This makes it fundamentally different from the Magnitude Stroop where both dimensions are purely numerical. More closely related is the Symbolic-Nonsymbolic Stroop task [18]. The latter uses adjacent arrays of numerical symbols of different length (e.g., 4444 vs 333). Participants are asked to indicate the side containing either the greater quantity of symbols (nonsymbolic task) or the numerically larger symbol (symbolic task). In this sense, Symbolic-Nonsymbolic Stroop task also manipulates magnitudes and numerosity, however, the Magnitude Stroop allows for more degrees of freedom in terms of both a greater range of stimuli and differences between symbolic and non-symbolic representations. Moreover, it is strongly agreed that enumeration of small and large arrays differs greatly (pg.1053) [7], which complicates comparisons between trials in the Symbolic-Nonsymbolic Stroop task.

Several variations of the Stoop task involving numbers have been developed because investigating potential differences in how people estimate magnitudes is a crucial topic across different disciplines. Neural evidence over the last years suggests that the underlying representation of numerical information is abstract independently of the modality with which stimuli are represented [19]. However this is a contentious point, as some studies have found differences (or a tendency towards a difference) between modalities (e.g., digits, verbal numbers, numerosity, [10,20,21]), so this is still an open debate. Crucially, we contribute to this literature by proposing a new method which aims to investigate processes interference created by the different modalities the numerical quantities are represented.

How we estimate magnitudes is incredibly important also for economic and financial decision making. There is growing evidence that even relatively complicated economic choices involving decisions between risky prospects are susceptible to how magnitudes, i.e., numbers, are represented and are heavily skewed by psychophysical laws [22–25]. These recent results replicate the original experiments [26] which showed psychometric effects compatible with Webers’ laws typically found in magnitudes also in purely (symbolic) numerical representations (e.g., comparing the number 7 to the number 9). These studies suggest that the numbersense is a high-generalised system, capable of combining numerical information across different formats even in the context of relatively complicated (economic) choices, but it is unclear whether there is a difference in performance across different modalities of representing economically-relevant quantities, e.g., amounts of money.

Across two experiments, an online and an in-person study, we find a reliable Stroop effect for when people are asked to estimate the number of dots while ignoring the largest number (DOT condition). Instead, we find weak evidence for the presence of a Stroop effect when people are asked to select the largest number and ignore the number of dots (numerosity) of the stimuli (NUMBER condition). These results provide suggestive evidence for the idea that the numbersense across modalities has a shared representation.

## Methods

### Open practices statement

We obtained ethical approval from the Office of Research Ethics and Integrity of the University of Melbourne, we obtained digital, informed consent for Study 1 (online) and written, informed, witnessed consent for Study 2 (in person). The data and code can be found in OSF https://osf.io/hgrqs, DOI: https://doi.org/10.17605/OSF.IO/HGRQS. This research complies with the Declaration of Helsinki (2023) and the study was approved by the Ethics Commission of the University of Melbourne (30784). We pre-registered the studies at AsPredicted 201184 and 242616, and follow the pre-registration. Data collection for the first study took place between the 07/08/2025 and 09/08/2025. Data collection for the second study took place between the 01/09/2025 and 17/09/2025.

### Study design

The two studies had the same experimental design, with the only difference that Study 1 was performed online on Prolific while Study 2 was performed offline in a laboratory experiment. Participants performed the Magnitude Stroop task. The task presented participants with two stimuli each containing different amounts of dots. The dots in each stimulus were arranged to form Arabic numbers. Participants were presented with two blocks, one for each condition, each composed of 40 trials, for a total of 80 trials in the study. Which condition participants saw first was randomized between participants. In one condition (DOT) participants were asked to select the option with the largest number of dots and ignore the symbolic numbers. In the NUMBER condition, participants were asked to select the largest number and ignore the number of dots. Independently of the two conditions, sometimes the stimulus with the largest number of dots was not the one which was represented by the largest number, we define these trials as incongruent. Instances where the largest number was also the one containing more dots were classified as congruent trials. See Fig 1 for a visual representation of the two situations. See S1 File for the instructions participants received.

### Power

The sample size was determined based on a significance threshold of α=0.05, for a small effect size of *d* = 0.2, and to ensure a power of 0.8 for a non-parametric, within-subject test, which leads to a required sample of *N* = 156. Since the Stroop effects relies on response times, which are likely to be imprecisely measured in online studies, we pre-registered to collect *N* = 200 participants. Given the results of the first online study we updated the estimated sample size for Study 2 (offline) and estimated a required sample size of *N* = 60.

### Participants

We validated the proposed task in two experimental studies, Study 1 was an online experiment on Prolific for which we planned to recruit *N* = 200 participants from the general population and only excluded those who do not complete the entire study. Participants might not complete the task because they decided to stop or because their experience was terminated when they did not pass common attention checks which were unrelated to the content of the study. Study 2 (*N* = 60) was an offline experiment among the student population of the University of Melbourne. For both studies, we perform within-subjects, non-parametric tests (Wilcoxon signed-rank test, WSR) for differences between conditions.

Study 1 took about 7 minutes and comprise of 42.16% female participants with an average age of 36.42 (median 33, min 18, max 83). Subjects earned 11.69 Pounds per hour for their participation. Participants are highly educated with only 0.49% without a university degree, 54.90% with a bachelor, 23.53% with a master, and 3.92% with a doctorate degree. In Study 2 participants were students of the University of Melbourne. They took about 5 minutes to complete the task, which was part of a larger set of tasks not reported in this work. Subjects earned a flat fee of 6.5 USD for their participation in this latter study.

### Stimuli

Stimuli were constructed by arranging dots to form Arabic numerals. See the list of stimuli in Table A in S1 File. In particular, we used a 9×5 (rows per columns) grid to arrange the dots. Although the ranking of stimuli based on the number of dots is not affected by (reasonable) grid changes, the precise differences do depend on the number of columns and rows used to arrange dots. We designed the stimuli to have 60% incongruent trials, which were equally represented across different digits. That is, we presented trials with 1 digit numbers, but also 2,3, and 4 digits. This allowed us to explore potential differences in behaviour and investigate which implementation of the method is best. Other considerations were used to create the stimuli. We never had the same symbolic number on the left and on the right, in order to allow for short-cuts in estimating magnitudes and/or differences. No trials had the same number of dots between stimuli, in order to allow a correct answer to exist. All presented dots were of the same dimeter and all numbers covered the same surface area, but the density of the dots within a number was mechanically different across different Arabic numeral. Moreover, all dot quantities were in the “counting” range compared to the “subitizing” range [27,28]. The order of trials within each block was randomised across participants. We tested for potential order effects in the trial order, by adding a control-variable to the regression analyses, and found no statistically-significant effect regarding error rates and in the distribution of response times. We randomised the left-right position of stimuli to balance their presentation. The proportion of incongruent trials was set to 60% as a compromise between maximising the number of interesting (incongruent) trials and the result reported in the literature that a higher the proportion of incongruent trials reduced the incongruency effect [29].

### Statistical analysis

Our main dependent variable of interest is the Stroop effect. This is the difference in the distribution of response times for correct answers between incongruent and congruent trials. We are interested in whether there is a Stroop effect and whether it is larger or smaller in the NUMBER compared to the DOT condition. Note that we compare the magnitude of the Stroop effects within subjects, hence mechanical differences in response times distribution due to differences in tasks and heterogeneity in the quality of the recorded response times between participants should cancel out. Moreover, due to the within-subject analysis absolute differences in response times between conditions also do not matter. Further note that we do not compare differences in response times between the NUMBER and the DOT condition directly, as they are expected to present mechanically different distributions. We instead are interested in differences in the magnitude of the Stroop effect, i.e., differences in response times of correct choices between congruent and incongruent trials within each condition, which should account for some of the mechanical differences between tasks.

A secondary variable of interest is the performance across conditions. That is, the proportion of correct choices in congruent vs. incongruent trials. Although error rates in the Stroop task tend to be small, we might be able to observe differences between conditions because the Magnitude Stroop task is more complex compared to the standard Stroop task.

Statistical differences in the dependent variables of interest between conditions are investigated using non-parametric within-subject (WSR) tests as well as random-effects linear (and probit) regressions. The latter approach considers response times for correct choices (the probability of a correct choices) as the dependent variable, with a dummy for incongruent trials further dividing the dataset based by condition (i.e., DOT vs. NUMBER). Lastly, we explore differences in efficiency of responses (speed-accuracy trade-off) using regression analyses. From all response time analyses we exclude outliers, which are defined as 2 standard deviations away from the individual mean.

## Results

### Performance

**Fig 2:**
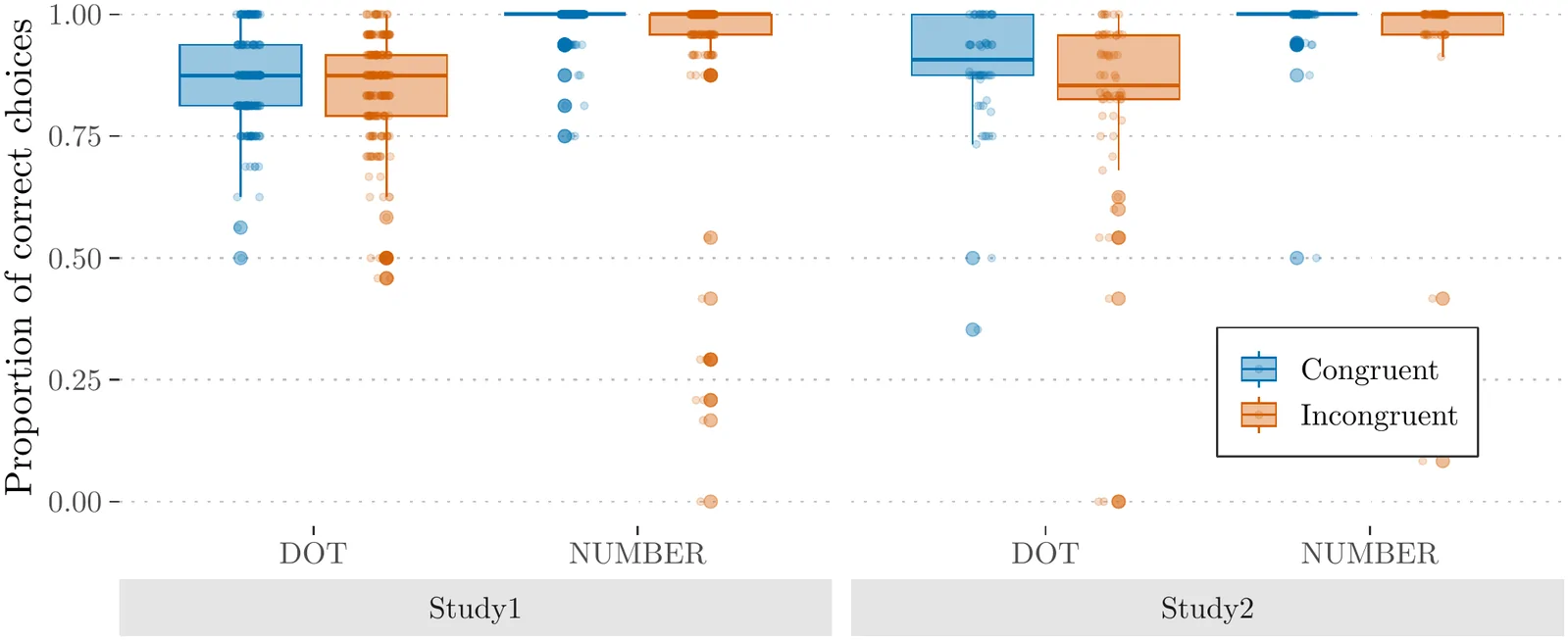
Distribution of error rates conditional on trial type (congruent vs. incongruent) and condition (DOT vs. NUMBER) for Study 1 (left panel) and Study 2 (right panel).

In Study 1, in the DOT condition the average proportion of correct answers is 85.56% which is smaller than the proportion of correct choices in the NUMBER condition 96.67% (Wilcoxon signed-rank test, WSR: N=204,z=−10.447,p<0.0001, effect size, Cohen’s *d* = 0.889). We find a similar effect in Study 2, which was in person compared to online (proportion of correct answers: DOT 85.28% vs. NUMBER 97.12%; WSR: N=60,z=−6.224,p<0.0001, effect size = 0.866). As Fig 2 shows, and as expected, performance is higher for congruent than incongruent trials in both conditions and both studies. In particular, in Study 1, under the DOT condition the proportion of correct choices in congruent trials (86.58%) is significantly higher than the one in incongruent trials (84.87%; WSR: *N* = 204, *z* = 1.779, *p* = 0.038, effect size = 0.866). We find a similar effect for the NUMBER condition (Proportion of correct answers: congruent 98.90% vs. incongruent 95.18%; WSR: *N* = 204, *z* = 5.577, *p* < 0.0001, effect size = 0.288). Study 2 qualitatively replicates the results with a larger proportion of correct choices in congruent than incongruent trials across conditions (DOT, proportion of correct answers: congruent 89.10% vs. incongruent 82.76%; WSR: *N* = 60, *z* = 2.512, *p* = 0.011, effect size = 0.280; NUMBERS: congruent 98.35% vs. incongruent 96.31%; WSR: *N* = 60, *z* = 2.359, *p* = 0.019, effect size = 0.179).

### Response times

**Fig 3:**
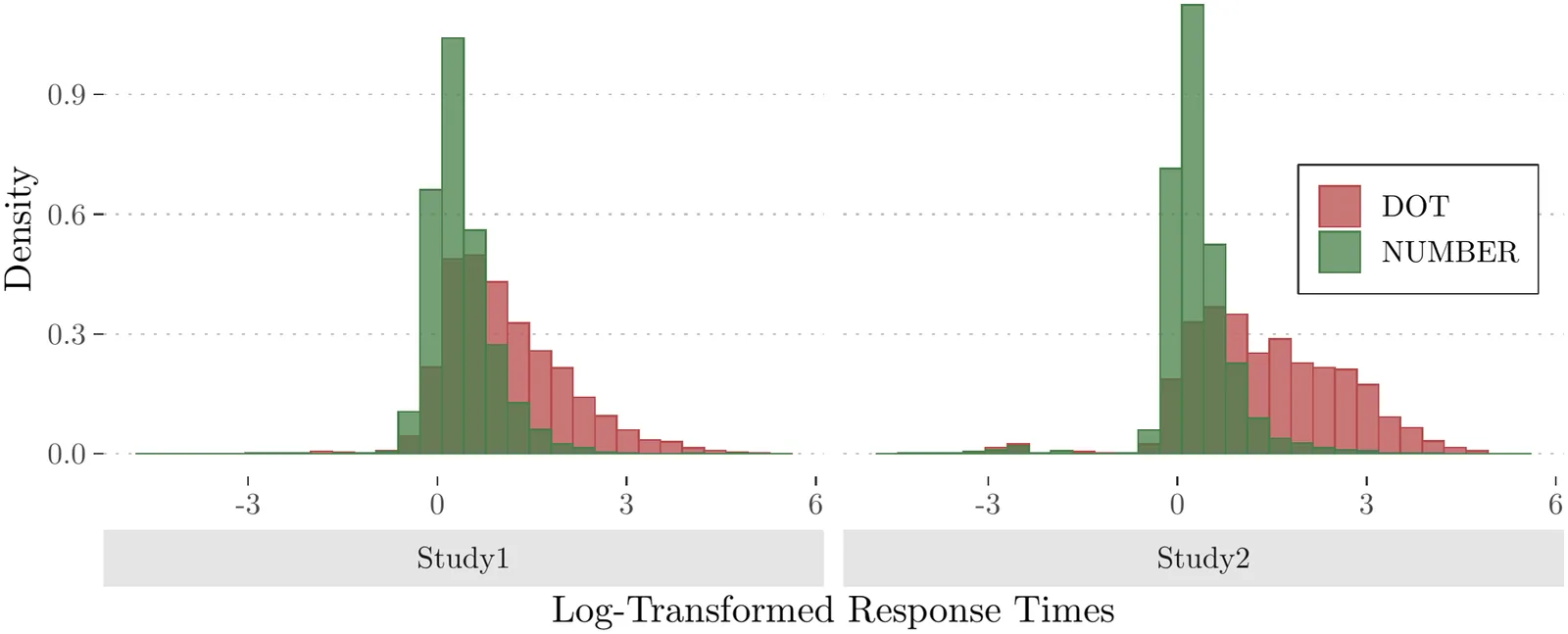
Distribution of (log-transformed)individual-average response times by condition (DOT vs. NUMBER) for Study 1 (left panel) and Study 2 (right panel).

As Fig 3 shows, participants took on average longer in the DOT (Study 1: 4.236 s, Study 2: 6.809) than NUMBER condition (Study 1: 1.632 s, WSR test: *N* = 204, *z* = 11.882, *p* < 0.001, effect size 0.550; Study 2: 1.627 s, WSR test: *N* = 60, *z* = 6.706, *p* < 0.0001, effect size 0.904), this is due to the fact that in the DOT conditions participants are asked to estimate the number of dots which takes more time than just evaluating which of the two presented numbers is larger.

**Fig 4:**
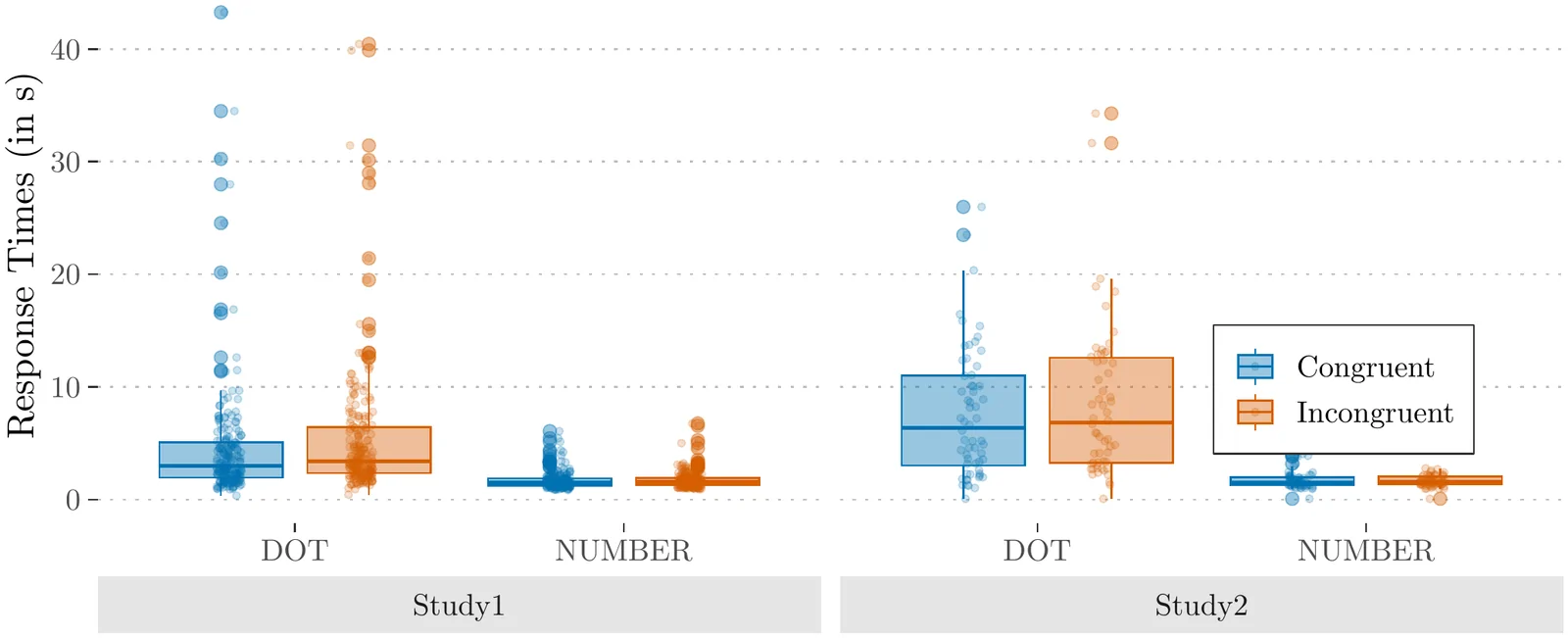
Distribution of individual-average response times for correct answers conditional on trial type (congruent vs. incongruent) and condition (DOT vs. NUMBER) for Study 1 (left panel) and Study 2 (right panel).

Fig 4 reports the distribution of response times by condition, trial type, and distinguishing correct choices from errors in the two studies. We observe a strong Stroop effect in the DOT condition. That is, longer response times of correct choices in incongruent compared to congruent trials (Study 1: incongruent 4.323 s vs. congruent 3.854 s, WSR test: *N* = 204, *z* = 7.742, *p* < 0.001, effect size 0.356; Study 2: incongruent 7.105 s vs. congruent 6.306 s, WSR test: *N* = 58, *z* = 2.698, *p* = 0.006, effect size 0.350). We do not observe a statistically significant difference in the distribution of response times of correct answers in the NUMBER condition between incongruent and congruent trials (Study 1: incongruent 1.621 s vs. congruent 1.622 s, WSR test: *N* = 203, *z* = 1.768, *p* = 0.077, effect size 0.002; Study 2: incongruent 1.629 s vs. congruent 1.586 s, WSR test: *N* = 60, *z* = 0.589, *p* = 0.561, effect size 0.096). In this sense, we observe a stronger Stroop effect for DOT (Study 1: 0.472 s, Study 2: 0.798 s) than NUMBER (Study 1: −0.001 s, WSR test: *N* = 203, *z* = 6.715, *p* < 0.001, effect size = 0.328; Study 2: 0.046 s, WSR test: *N* = 58, *z* = 2.288, *p* = 0.022, effect size = 0.328).

### Regression analyses

We now turn to panel regression analyses in order to account for subjects’ heterogeneity. Table 1 reports a random-effect panel probit regression on the probability of a mistake with robust standard errors clustered at the individual level. Model 1 and 2 reports trials in the DOT condition, while models 3 and 4 those in the NUMBER condition. Models 1 and 3 report the data for Study 1, while models 2 and 4 do the same for Study 2. Across all models of Table 1, we observe more errors in incongruent compared to congruent trials, which replicates the results obtained by the individual tests.

**Table 1:** Random effect probit regression on the probability of a mistake.

Error	DOT		NUMBER	
	Study 1	Study 2	Study 1	Study 2
	Model 1	Model 2	Model 3	Model 4
Incongruent	0.071**	0.293***	0.949***	0.518***
	(0.036)	(0.070)	(0.101)	(0.166)
Constant	−1.141***	−1.346***	−3.400***	−3.051***
	(0.034)	(0.086)	(0.154)	(0.252)
Log Lik.	−3329.553	−924.986	−760.649	−202.649
Observations	8160	2400	8160	2400
Subjects	204	60	204	60

*Notes:* Robust standard errors in brackets, * *p* < 0.1, ** *p* < 0.05, *** *p* < 0.01.

Table 2 reports the analysis of log-transformed response times of correct answers using a panel-random effect regression with robust standard errors clustered at the individual level. The structure of this table is the same as Table 1. Across all models, the regression analyses on response times broadly reproduces the individual tests reported above. The base category is correct answers in congruent trials. The estimated coefficient for the dummy variable *Incongruent* in Model 1 provides the statistical test for the Stroop effect (slower response times of correct answers in incongruent than congruent trials). We find support for a significant presence of the Stroop effect for the DOT condition, but not for the NUMBER condition, as the latter is only marginally significant (*p* < 0.05, Model 3, Study 1).

**Table 2:** Random effect regression on log-transformed response times for correct answers.

Log.RT	DOT		NUMBER	
	Study 1	Study 2	Study 1	Study 2
	Model 1	Model 2	Model 3	Model 4
Incongruent	0.176***	0.123***	0.021**	0.014
	(0.017)	(0.039)	(0.010)	(0.019)
Constant	0.950***	1.304***	0.361***	0.291***
	(0.046)	(0.114)	(0.021)	(0.061)
*R* ^2^	0.009	0.005	0.001	0.001
Observations	6981	2043	7888	2336
Subjects	204	60	204	60

*Notes:* Robust standard errors in brackets, * *p* < 0.1, ** *p* < 0.05, *** *p* < 0.01.

Table B in S1 File reports the analysis of response times without separating for tasks. This allow us to investigate whether the observed asymmetry arises from a difference in the overall processing time across tasks. The results are qualitatively unchanged compared to those reported in Table 2, i.e., we still observe an asymmetry (Stroop effect) for DOT but not for the NUMBER condition, even when we analyse the two tasks together. These results strengthen the idea that the observed asymmetry is not due to differences in the level of response times (and hence processing requirements) across conditions.

Table 3 reports the analysis of response times of the inverse efficiency score. The idea is to investigate differences in speed-accuracy trade-off across conditions (DOT vs. NUMBER) and depending on congruency. This allow us to integrate the investigation of accuracy and response times in a unique dependent variable. As indicated by [30] among others, the inverse efficiency score is calculated as the mean response time of the correct responses (in seconds) in a particular condition divided by the proportion of correct choices in that condition. The structure of this table is the same as Table 1.

**Table 3:** Random effect regression on inverse efficiency score.

Log.RT	DOT		NUMBER	
	Study 1	Study 2	Study 1	Study 2
	Model 1	Model 2	Model 3	Model 4
Incongruent	0.400***	0.750***	0.004	0.041
	(0.001)	(0.005)	(0.878)	(0.485)
Constant	3.799***	5.959***	1.616***	1.585***
	(0.002)	(0.001)	(0.001)	(0.001)
*R* ^2^	0.001	0.007	0.009	0.001
Subjects	204	60	204	60

*Notes:* Robust standard errors in brackets, * *p* < 0.1, ** *p* < 0.05, *** *p* < 0.01.

Across all models, the regression analyses on inverse efficiency score reproduces the individual tests and regression analyses reported above. The dummy variable *Incongruent* in Model 1 and Model 2 shows evidence for the Stroop effect (slower response times of correct answers in incongruent than congruent trials) for the DOT condition across both studies. However, we find no statistically significant Stroop effect for the NUMBER condition (Model 3 and 4). The results of the efficiency analysis suggest that the asymmetry we observe between conditions is unlikely to be explained by differences in response strategy (e.g., participants slowing down in the DOT condition to maintain accuracy under incongruent trials, whereas in the NUMBER task they may have responded more quickly at the cost of higher error rates), buy is rather more likely that the results derive from differences in representational overlap.

Some participants responded with below-chance accuracy (i.e., the proportion of correct choices is below 50%). This might be due to misunderstanding of instructions or uncooperative participants. Participants with more than 50% error rate are *N* = 8 for the online study and *N* = 4 in the offline study. Results are qualitatively unchanged when we exclude the potentially uncooperative or confused participants.

## Discussion

How we represent quantities is a fundamental and interdisciplinary question. Sometimes we need to decide which group of objects has more elements, but other times we compare symbolic numbers. In this work we investigated whether representing quantities as numbers compared to groups of objects share a common representation. We do so by validating the *Magnitude Stroop* task, which represents stimuli at the same time as symbolic numbers and arranged dots. In this task, participants are asked to decide sometimes which of the two stimuli has the largest number, and other times they should choose the option with more dots. In some trials the two rules agree, while in others not. Hence, the two dimensions with which stimuli are represented can interfere with each others.

We find strong evidence for asymmetrical processing of symbolic and non-symbolic numerical magnitudes. In particular, we show a robust Stroop effect, that is slower response times for correct choices in incongruent than congruent trials, when people are asked to choose which stimulus has more dots, but not when they are asked which one is the largest number. This is further linked to a difference in performance and efficiency (speed-accuracy tradeoff), with accuracy higher in congruent trials (when the rules agree) than incongruent ones. In this sense, our findings are compatible with the idea that our numbersense processes symbolic and non-symbolic magnitudes at least partially in parallel or simultaneously. These results align with previous similar findings showing a relation between how symbolic and the non-symbolic numerical magnitudes are represented [6,18,19]. Moreover, we show that the symbolic representation of magnitudes involve less conflict resolution than non-symbolic representations, i.e., present a stronger Stroop effect when people are asked to ignore the symbolic representation compared to ignoring the non-symbolic one.

However, these results should be take with some caveats, as previous findings have also shown that the processing of symbolic numerals can be dissociated from the one of the non-symbolic numerosities [31]. In this sense, more than the empirical results of this work, the main contribution of this work is the development and validation of a new method to investigate the relation between how symbolic and non-symbolic numerosities. Specifically, given the peculiar properties of the Magnitude Stroop task, which allows to manipulate at the same time and across very similar dimensions symbolic and non-symbolic numerosities, it could allow to shed light and progress the debate over the identity or dissociation of how numerosities are processed.

The present study presents some limitations. While all dots were the same diameter, colour, and brightness, and all numbers covered the same surface area, the density of the dots across different stimuli was mechanically different. These visual properties of the stimuli have been shown to influence quantity estimation [32] and hence might have played a role in the obtained results. Further, in some of the stimuli pairs the leftmost digit was different. This potentially allowed participants to use simplified strategies, in the NUMBER trials, i.e., only looking at the left-most digit.

Another consideration that should be highlighted is that the conditions might not entail the same level of difficulty. That is, depending on the metric used to compare the symbolic representations or the dot-based comparisons, one could argue the first are much farther apart than the second, and this mismatch is more severe as digit length increases (see Table A in S1 File for the list of stimuli). This might provide a plausible explanation for the observed difference in accuracy overall response time distribution between conditions (DOT vs. NUMBER). In particular, regarding the difference in distribution of response times, participants may have used different strategies. For example, some may have counted each dot, leading to relatively slow responses. In such cases, there would likely be limited to no interference in the NUMBER condition. However, other participants may have approximately compared the left and right stimulus, leading to comparatively faster responses. Only for these latter participants we would predict to observe interference. In this sense, the presence of a mixture of these strategies in the NUMBER condition could explain why the Stroop effect was small or even absent. While this consideration does not invalidate the presence of Stroop-like effects, it might moderate its effects, and future studies should further investigate these effects in details by manipulating the range (and hence difficulty) within the two conditions.

Generally, the performance observed in this work is lower than in previous experiments [12,13] both in the online and in person studies. This might be due to the fact that the presented stimuli are more complex than standard Stroop experiments, i.e., number and magnitude comparisons vs. one-word comparisons. Nevertheless we observe, as expected, a significant difference between incongruent and congruent trials, and performance close to the ceiling, indicating that participants were paying attention and understood the tasks.

While these results suggest that indeed there are some overlaps between how we represent magnitudes across modalities, still much is unclear about the mechanisms. However, the proposed Magnitude Stroop task might help future investigations in this direction. Further, this study presents some limitations with respect of how the reported results should be interpreted. For example, tasks which involve the estimation of magnitudes are usually implemented with randomly arranged dots and with a very large number. In this work the dots are specifically arranged to form Arabic numbers, which might have facilitated participants job. In this sense, we deviate from previous studies in both how the stimuli are represented and on the scale of their magnitude. In this sense, we contribute to the debate about asymmetric processing of numerical and nonnumerical (symbolic) magnitudes by providing a new method to further our understanding of these issues.

## Supporting information

S1 File Supplementary information. Contains the full participant instructions (Section 1), the complete list of stimuli used in both studies (Table A, Section 2), and robustness analyses including the pooled response-time regression (Table B, Section 3).
(PDF)
