# Supplementary material for: How do we estimate magnitudes? Validating the magnitude stroop task
Source: PLoS One. 2026 Aug 31;21(8):e0357108. doi: 10.1371/journal.pone.0357108 (PMC13528955; doi:10.1371/journal.pone.0357108)
Supplement: Supplementary file 1 [file pone.0357108.s001.pdf]

SUPPORTING INFORMATION FOR:
How Do We Estimate Magnitudes? Validating the Magnitude Stroop Task

Michele Garagnani, Nitin Yadav

1 Instructions

The following instructions were provided to participants in both studies.

[**Description of the study**]

In this study we will ask you to compare numbers. The study is expected to last 10 minutes. You can participate in this study only using a computer, no mobile or tables are allowed.

[**Instructions**]

Thank you for participating in this study. This study will take approximately 10 minutes to complete. You will be presented with 80 choices between two options. These options will appear as numbers dislayed side by side, one on the left and one on the right.

Each number is represented by a collection of dots, with the quantity of dots varying for each number.

Your task is to select one of the two numbers based on a specific rule. Please not that the rule will change at different points during the study.

To select the left number press the left-arrow key, and to select the right number press the right-arrow key.

Please press the right arrow key to start the task.

[**NUMBER condition**]

In the next series of questions:
Choose the option with the largest number (ignore the number of dots).
Press the right arrow to start.

[**DOT condition**]

In the next series of questions:
Choose the option with more WHITE dots (ignore the numbers).
Press the right arrow to start.

2 List of stimuli

Table 1: List of stimuli.

ID Pair	Left Stimulus	Left Numb. of Dots	Right Stimulus	Right Numb. of Dots	Congruent
1	2	21	7	13	0
2	3	21	1	9	1
3	5	21	0	27	0
4	6	24	4	17	1
5	7	13	3	21	0
6	37	34	24	38	0
7	46	41	51	30	0
8	52	42	69	48	1
9	87	37	91	33	0
10	96	48	27	34	1
11	294	62	358	66	1
12	316	54	257	55	0
13	631	54	572	55	0
14	792	58	145	47	1
15	917	46	345	59	0
16	2049	89	8513	75	0
17	3082	93	6459	86	0
18	4920	89	8531	75	0
19	8230	93	5964	86	1
20	9016	84	2375	76	1

3 Robustness Analyses

Table 2 reports the analysis of log-transformed response times of correct answers using a panel-random effect regression with robust standard errors clustered at the individual level. The structure of this table is the same as Table 1 and Table 2. The difference with Table 2 is that we do not analyse separately the two conditions (DOT and NUMBER), but investigate them together using an interaction term. Across all models, the regression analyses qualitatively reproduces the results of Table 2.

Table 2: Random effect regression on log-transformed response times for correct answers.

Log.RT	Study 1	Study 2
Incongruent	0.015	0.005
	(0.015)	(0.032)
DOT	0.605***	1.041***
	(0.016)	(0.036)
Incongruent × DOT	0.161***	0.143***
	(0.021)	(0.047)
Constant	0.360***	0.286***
	(0.026)	(0.079)
R^2	0.194	0.293
Observations	14869	4379
Subjects	204	60
